# Supplementary material for: Deletion of wfs1 Impairs Oligodendrocyte Precursor Cells Dorsal Distribution and Myelination Through the wfs1-hmgcs1 Axis in Zebrafish
Source: Biology (Basel). 2026 Aug 16;15(16):1402. doi: 10.3390/biology15161402 (PMC13510151; doi:10.3390/biology15161402)
Supplement: Supplementary file 1 [file biology-15-01402-s001.zip › File S1-Transcriptome sequencing data and differentially expressed genes (DEGs) in wfs1-- zebrafish at 5 dpf/2.KEGG/wfs1KOvsWT/GSEA/wfs1KO/TYROSINE_METABOLISM/TYROSINE_METABOLISM(DRE00350).html]

Details for gene set TYROSINE\_METABOLISM(DRE00350)[GSEA]

|  || Dataset | deg.sample |
| Phenotype | sample.cls |
| Upregulated in class | wfs1KO |
| GeneSet | TYROSINE\_METABOLISM(DRE00350) |
| Enrichment Score (ES) | 0.3713425 |
| Normalized Enrichment Score (NES) | 1.4518055 |
| Nominal p-value | 0.0734127 |
| FDR q-value | 0.17401266 |
| FWER p-Value | 0.807 |
Table: GSEA Results Summary

  

Fig 1: Enrichment plot: TYROSINE\_METABOLISM(DRE00350)      
 Profile of the Running ES Score & Positions of GeneSet Members on the Rank Ordered List

  

| PROBE | DESCRIPTION (from dataset) | GENE SYMBOL | GENE\_TITLE | RANK IN GENE LIST | RANK METRIC SCORE | RUNNING ES | CORE ENRICHMENT || 1 | 116517 | adh5 |  |  | 148 | 4.502 | 0.0977 | Yes |
| 2 | 445287 | hpdb |  |  | 629 | 2.726 | 0.1446 | Yes |
| 3 | 30505 | dbh |  |  | 690 | 2.615 | 0.2021 | Yes |
| 4 | 541429 | aoc2 |  |  | 862 | 2.303 | 0.2491 | Yes |
| 5 | 561410\_1 | tat |  |  | 1121 | 1.974 | 0.2859 | Yes |
| 6 | 394142 | hpda |  |  | 1435 | 1.701 | 0.3147 | Yes |
| 7 | 406688\_1 | got2a |  |  | 2167 | 1.300 | 0.3213 | Yes |
| 8 | 565370\_1 | comtb |  |  | 2427 | 1.198 | 0.3404 | Yes |
| 9 | 100331172 | epx |  |  | 2649 | 1.129 | 0.3591 | Yes |
| 10 | 561372 | comta |  |  | 3009 | 1.034 | 0.3713 | Yes |
| 11 | 565370 | comtb |  |  | 4234 | 0.788 | 0.3508 | No |
| 12 | 558050 | si:ch211-127i16.2 |  |  | 4370 | 0.766 | 0.3640 | No |
| 13 | 100332609 | LOC100332609 |  |  | 5295 | 0.647 | 0.3497 | No |
| 14 | 404730 | mao |  |  | 5766 | 0.610 | 0.3488 | No |
| 15 | 30384 | th |  |  | 6648 | 0.524 | 0.3330 | No |
| 16 | 402939 | zgc:77938 |  |  | 7452 | 0.457 | 0.3182 | No |
| 17 | 414844 | th2 |  |  | 7591 | 0.448 | 0.3240 | No |
| 18 | 406330 | got1 |  |  | 8454 | 0.406 | 0.3062 | No |
| 19 | 751093 | mif |  |  | 8471 | 0.405 | 0.3149 | No |
| 20 | 322482 | adh8a |  |  | 8920 | 0.373 | 0.3093 | No |
| 21 | 110437932 | LOC110437932 |  |  | 9193 | 0.353 | 0.3088 | No |
| 22 | 436754 | gstz1 |  |  | 11729 | 0.204 | 0.2338 | No |
| 23 | 402841\_1 | adh8b |  |  | 11968 | 0.193 | 0.2307 | No |
| 24 | 791730 | got1l1 |  |  | 13108 | 0.135 | 0.1980 | No |
| 25 | 406330\_1 | got1 |  |  | 14060 | 0.089 | 0.1702 | No |
| 26 | 570457 | aox6 |  |  | 15519 | 0.021 | 0.1249 | No |
| 27 | 402841 | adh8b |  |  | 16797 | -0.026 | 0.0854 | No |
| 28 | 30505\_1 | dbh |  |  | 17052 | -0.035 | 0.0782 | No |
| 29 | 335974 | got2b |  |  | 18318 | -0.090 | 0.0405 | No |
| 30 | 561410 | tat |  |  | 20583 | -0.193 | -0.0262 | No |
| 31 | 282559 | aldh3b1 |  |  | 20742 | -0.200 | -0.0266 | No |
| 32 | 406651 | ddc |  |  | 20753 | -0.201 | -0.0224 | No |
| 33 | 437022 | tyrp1b |  |  | 22480 | -0.295 | -0.0699 | No |
| 34 | 259189 | hgd |  |  | 22562 | -0.299 | -0.0656 | No |
| 35 | 30384\_1 | th |  |  | 23375 | -0.353 | -0.0831 | No |
| 36 | 100333145 | tyrp1a |  |  | 23687 | -0.372 | -0.0844 | No |
| 37 | 557008 | aldh3b2 |  |  | 24033 | -0.394 | -0.0863 | No |
| 38 | 58074 | dct |  |  | 25044 | -0.450 | -0.1078 | No |
| 39 | 30207 | tyr |  |  | 25585 | -0.492 | -0.1136 | No |
| 40 | 558050\_1 | si:ch211-127i16.2 |  |  | 26135 | -0.540 | -0.1185 | No |
| 41 | 337166 | si:dkey-40g16.6 |  |  | 26277 | -0.554 | -0.1104 | No |
| 42 | 322372 | fah |  |  | 26329 | -0.559 | -0.0993 | No |
| 43 | 553762 | fahd1 |  |  | 27687 | -0.699 | -0.1260 | No |
| 44 | 64265 | aox5 |  |  | 27764 | -0.711 | -0.1122 | No |
| 45 | 116517\_1 | adh5 |  |  | 28219 | -0.775 | -0.1089 | No |
| 46 | 445287\_1 | hpdb |  |  | 29361 | -1.018 | -0.1216 | No |
| 47 | 406688 | got2a |  |  | 30634 | -1.617 | -0.1248 | No |
| 48 | 393427 | zgc:63568 |  |  | 31073 | -2.050 | -0.0919 | No |
| 49 | 569363 | tpo |  |  | 31142 | -2.138 | -0.0455 | No |
| 50 | 541429\_1 | aoc2 |  |  | 31543 | -3.032 | 0.0109 | No |
Table: GSEA details [plain text format]

  

Fig 2: TYROSINE\_METABOLISM(DRE00350)      
 Blue-Pink O' Gram in the Space of the Analyzed GeneSet

  

Fig 3: TYROSINE\_METABOLISM(DRE00350): Random ES distribution      
 Gene set null distribution of ES for **TYROSINE\_METABOLISM(DRE00350)**
